# Ecological niche modelling to estimate the distribution of *Culicoides*, potential vectors of bluetongue virus in Senegal

**DOI:** 10.1186/s12898-019-0261-9

**Published:** 2019-11-01

**Authors:** Mamadou Ciss, Biram Biteye, Assane Gueye Fall, Moussa Fall, Marie Cicille Ba Gahn, Louise Leroux, Andrea Apolloni

**Affiliations:** 1 Institut Sénégalais de Recherches Agricoles/Laboratoire National de l’Elevage et de Recherches Vétérinaires, BP 2057, Dakar-Hann, Senegal; 2 Laboratoire d’Ecologie Vectorielle et Parasitaire, Département de Biologie Animale, Faculté des Sciences et Techniques, Université Cheikh Anta Diop, Dakar, Senegal; 3 CIRAD, UPR AIDA, Dakar, Senegal; 4 AIDA, Univ Montpellier, CIRAD, Montpellier, France; 5 CIRAD, UMR ASTRE, 34398 Montpellier, France

**Keywords:** Vector-borne diseases, Afrotropical region, *Culicoides*, Bluetongue, Ecological modelling, MaxEnt, Boosted Regression Tree, Ecological Niche Factor Analysis, Suitable habitats

## Abstract

**Background:**

Vector-borne diseases are among the leading causes of morbidity and mortality in humans and animals. In the Afrotropical region, some are transmitted by *Culicoides*, such as Akabane, bluetongue, epizootic haemorrhagic fever and African horse sickness viruses. *Bluetongue virus* infection has an enormous impact on ruminant production, due to its high morbidity and mortality rates.

**Methods:**

A nationwide *Culicoides* trapping campaign was organized at the end of the 2012 rainy season in Senegal. A Maximum Entropy approach (MaxEnt), Boosted Regression Tree (BRT) method and Ecological Niche Factor Analysis (ENFA) were used to develop a predictive spatial model for the distribution of *Culicoides*, using bio-climatic variables, livestock densities and altitude.

**Results:**

The altitude, maximum temperature of the warmest month, precipitation of the warmest quarter, mean temperature of the wettest quarter, temperature seasonality, precipitation of the wettest quarter and livestock density were among the most important factors to predict suitable habitats of *Culicoides*. *Culicoides* occurrences were, in most of the cases, positively correlated to precipitation variables and livestock densities; and negatively correlated to the altitude and temperature indices. The Niayes area and the *G*roundnut basin were the most suitable habitats predicted.

**Conclusion:**

We present ecological niche models for different *Culicoides* species, namely *C. imicola*, *C. oxystoma*, *C. enderleini* and *C. miombo*, potential vectors of bluetongue virus, on a nationwide scale in Senegal. Through our modelling approach, we were able to determine the effect of bioclimatic variables on *Culicoides* habitats and were able to generate maps for the occurrence of *Culicoides* species. This information will be helpful in developing risk maps for disease outbreaks.

## Background

Vector-borne diseases are among the leading causes of morbidity and mortality in humans and animals. In the Afrotropical region, *Culicoides* species are the main vectors for highly devastating viruses such as Akabane, bluetongue (BT), epizootic haemorrhagic fever and African horse sickness (AHS) [1, 2]. BT, AHS and EHD are listed among the reportable diseases of the World Organization for Animal Health (OIE). Bluetongue virus (BTV) is transmitted to hosts, both wild and domestic ruminants, by the bites of midges of the genus *Culicoides*, and infections can lead to the host’s death. Few studies exist on the epidemiological situation in Senegal. Two of them estimated sero-prevalence varying between 30 and 59% for cattle and sheep [3, 4]. Understanding the trophic behaviour and the spatial dynamics of *Culicoides* species could help in controlling the spread of BT.

Studies on the trophic behaviour of *Culicoides* species have shown that these midges feed on various hosts in Afrotropical region, but mainly on mammals and birds [5–7]. The frequency of blood meals is 3 to 5 days depending on the availability of hosts, which are necessary to complete their gonotrophic cycle [8, 9]. After egg maturation, which occurs 2 to 4 days after the blood meal [10], females seek oviposition sites to deposit their eggs. The number of eggs laid varies from 30 to 250. A vermiform larva is free of pseudopods within 3 to 10 days after being hatched [11]. Larvae of *Culicoides* species live in various habitats, but they are mostly wet and enriched in organic matter of animal or plant origin [12–17].

Adult ecology can be studied taking a purely statistical approach. Predictive modelling of species geographical distributions, based on environmental conditions, is a major technique in analytical biology, with applications in conservation and reserve planning, ecology, evolution, epidemiology, invasive-species management and other fields [18–21]. Sometimes both presence and absence data are available for developing models, in which case general-purpose statistical methods can be used [22, 23]. However, whilst presence data can be collected through trapping campaigns, absence data are rather more difficult to collect and interpret.

Species distribution models (SDM) can be used to predict the distribution of species. Several methods, belonging to different classes, can be used to evaluate SDM:”profile” such as Domain [24], Mahalanobis distance [25], regression such as Generalized Additive Models (GAM) [26, 27]; machine learning such as Random Forest [28], Support Vector Machines (SVM) [29], Boosted Regression Trees [30], MaxEnt [31].SDM are used in a large selection of topics: forest landscape [32], wetland distribution [33], coastal benthic biodiversity [34], medicine [35], aquatic invasive species [36, 37].

In earlier work, Diarra et al. [23] modelled the spatial distribution of five *Culicoides* species of veterinary interest using two statistical approaches: a generalized linear model (GLM) with Poisson distribution, and a random forest (RF) model. The choice of the species (*C. imicola*, *C. oxystoma*, *C. enderleini*, *C. bolitinos and C. miombo*) was justified by their vectorial competence for the BTV and AHS viruses [38–41].

In this study, we combined an Ecological Niche Factor Analysis (ENFA) [42, 43] and species distribution modelling. We used an ENFA to select variables contributing to the ecological niche. The main advantage of ecological niches models, compared to other traditional regression modelling approaches, is that they require only presence data [44] and they effectively assess the likelihood of species presence, or the relative ecological suitability of a spatial unit, within the study area [45].

We then used Boosted Regression Trees and MaxEnt to predict species distribution, and compared their results. These two methods are widely used species distributions models for *Culicoides* distribution [46] and for vector-borne diseases such as Rift Valley Fever (FVR) [47–50], Trypanosomosis [51, 52], Chikungunya [53, 54], Japanese Encephalitis Virus (JEV) [55, 56], Malaria [57–61], Epizootic Haemorrhagic Disease (EHD) [62], Dengue [63–65] and Plague [66, 67].

Our work completes that of Diarra et al. [23] investigating the potential effect of bioclimatic variables and livestock densities to predict spatial distribution for four *Culicoides* species that are potential vectors of BTV (*C. imicola, C. oxystoma*, *C. enderleini and C. miombo*), and to identify the most suitable habitats in Senegal.

## Results

In all 1,373,929 specimens of the genus *Culicoides* belonging to at least 32 different species [23] were collected from 96 of the 108 sites visited at the end of the 2012 rainy season (in September and October). *C. oxystoma*, *C. enderleini*, *C. imicola* and *C. miombo* were the four most abundant species out of the species of veterinary interest [23]. For the 96 sites visited, *C. oxystoma* was present in 91 (94.79%), *C. enderleini* in 92 (95.83%), *C. imicola* in 92 (95.83%) and *C. miombo* in 77 (80.21%).

**Fig. 1:**
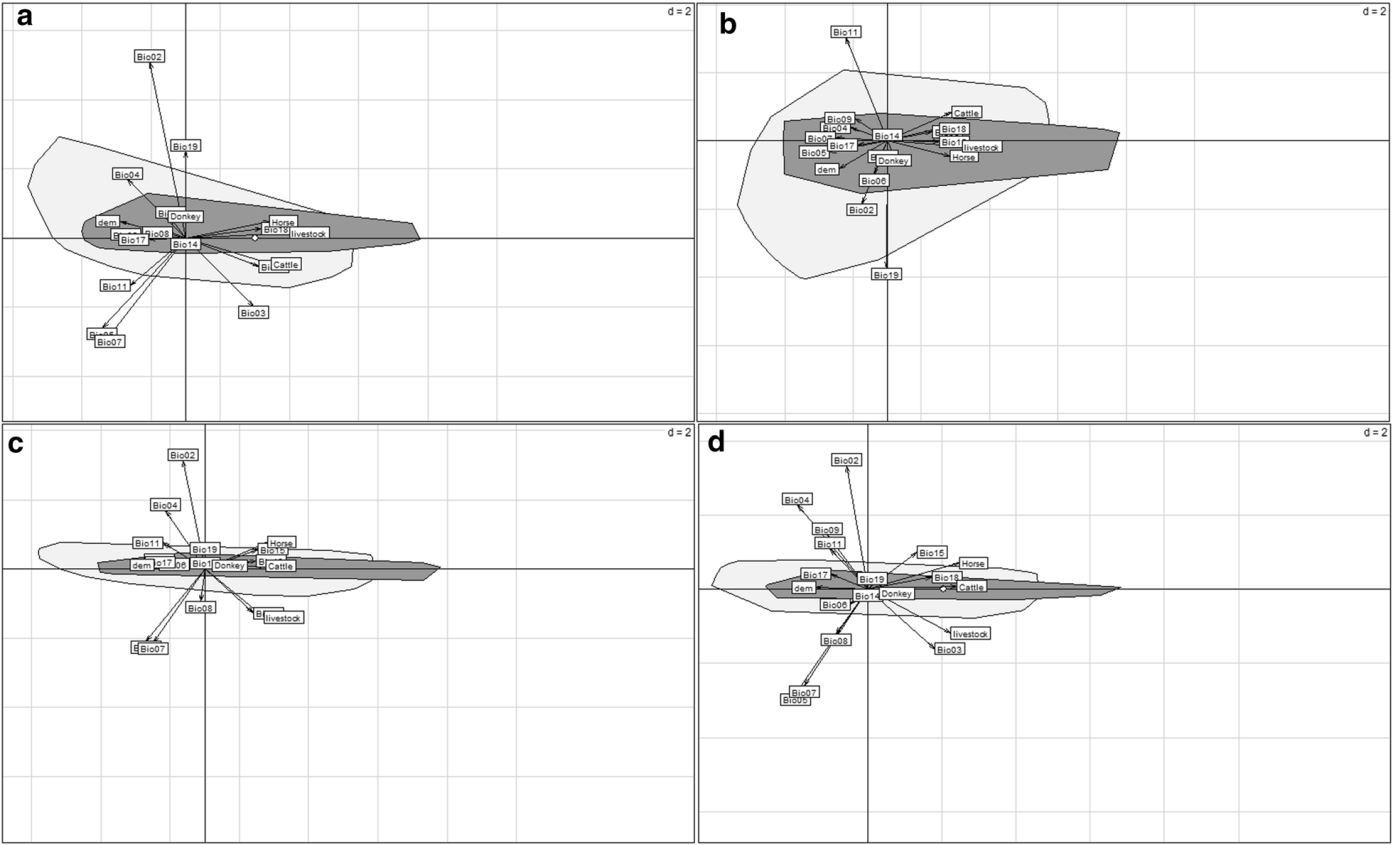
Ecological niche factor analysis (ENFA) of *Culicoides* distribution in Senegal. *C. imicola* (**a)**, *C. oxystoma* (**b**), *C. enderleini* (**c**) and *C. miombo* (**d**). Variables leading to ecological niche are represented into the light grey polygon and the dark grey polygon shows environmental conditions where *Culicoides* were observed (representation of the realized niche), and the small white circle corresponds to the barycentre of its distribution

The ENFA (Fig. 1) showed that the presence of BTV vectors was often positively correlated to some precipitation variables, such as the precipitation of the warmest quarter (Bio18) and precipitation seasonality (Bio15), and to most of the livestock (horses, cattle, donkeys, goats and sheep), either cumulated or taken separately (Fig. 1a–d). On the other hand, the altitude (dem) and most of the temperature indices were negatively correlated to species occurrence, notably the maximum temperature of the warmest month (Bio05), mean temperature of the wettest quarter (Bio08) and the annual temperature range (Bio07) (Fig. 1).

**Fig. 2:**
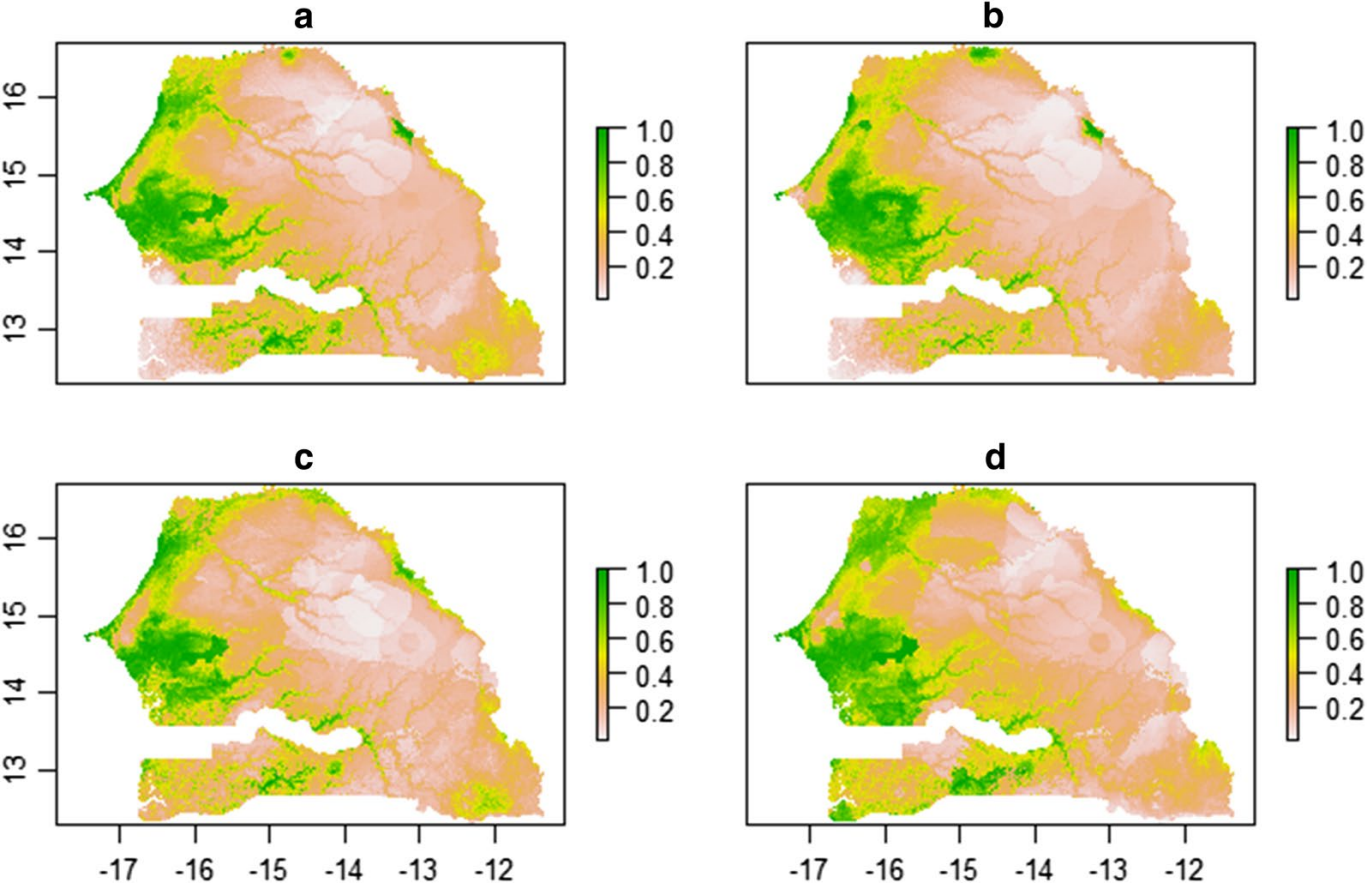
MaxEnt predicted suitable areas. *C. imicola* (**a**), *C. oxystoma* (**b**), *C. enderleini* **(c)** *and C. miombo* (**d**). Green areas indicate areas that are likely to have suitable habitats for this vector species, while lighter areas indicate areas that are less suitable for the vector

**Fig. 3:**
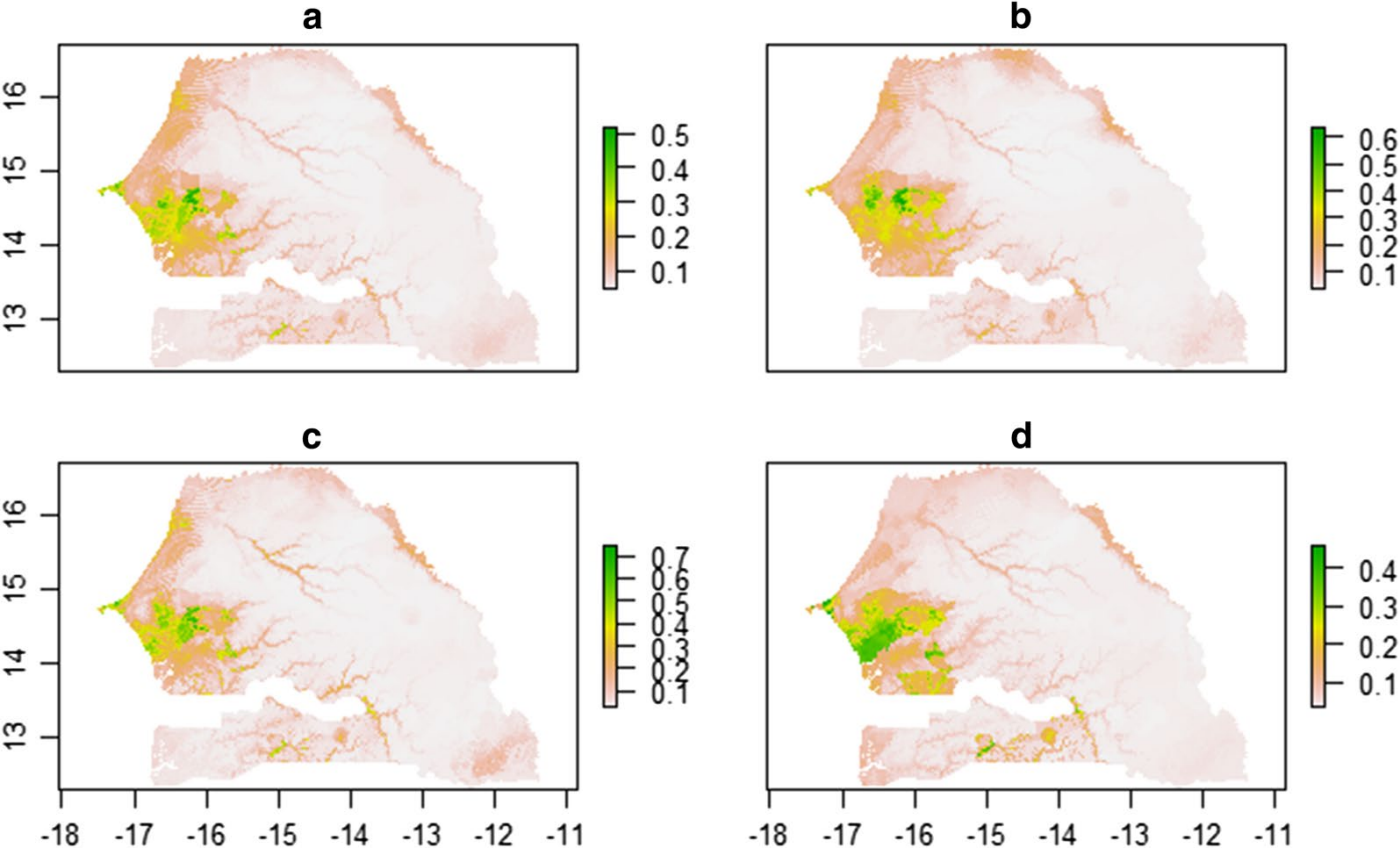
BRT predicted suitable areas. *C. imicola* (**a**), *C. oxystoma* (**b**), *C. enderleini* (**c**) and *C. miombo* (**d**). Green areas indicate areas that are likely to have suitable habitat for this vector species, while lighter areas indicate areas that are less suitable for the vector

For each species we informed the MaxEnt (Fig. 2) and BRT (Fig. 3) models with variables previously found in the ENFA, to predict their geographical distribution. The resulting maps, showed the predicted geographical distributions of these species based on the habitat suitability for each of the four species. The green areas shown are those of greater relative probability of occurrence, while lighter coloured areas are those where the relative probability of occurrence was slight or null. For the MaxEnt model, a high probability of species presence was predicted in the Niayes area and the Groundnut Basin. The Niayes area and northern zone were predicted to be favourable for *C. imicola* (Fig. 2a), *C. oxystoma* (Fig. 2b), *C. enderleini* (Fig. 2c). For *C. imicola, C. oxystoma, C. enderleini* and *C. miombo* (Fig. 2d) the predicted presence probabilities were high from the northern Gambia to the eastern Senegal. In the southern Senegal, all species were predicted to be present. The probability of species occurrence was low in the Ferlo area and southeastern area. For each species, the corresponding niche model has an Area Under the Curve (AUC) greater than 0.77 (Table 1): 0.821 for *C. imicola*, 0.773 for *C. oxystoma*, 0.823 for *C. enderleini* and 0.785 for *C. miombo*.

**Table 1:** Accuracy of the Niche Models: Area Under the Curve (AUC) for the MaxEnt and BRT models

Species	MaxEnt AUC	BRT AUC
*C. imicola*	0.821	0.813
*C. oxystoma*	0.773	0.817
*C. enderleini*	0.823	0.793
*C. miombo*	0.785	0.779

Comparatively to the MaxEnt model, the BRT model showed a similar predictive area for the ecological niche (Fig. 3). However, the probabilities of presence predicted by the BRT model were lower than those predicted by the MaxEnt model. The AUC values of the four species were greater than 0.77 (Table 1): 0.813 for *C. imicola*, 0.817 for *C. oxystoma*, 0.793 for *C. enderleini* and 0.779 for *C. miombo*.

**Fig. 4:**
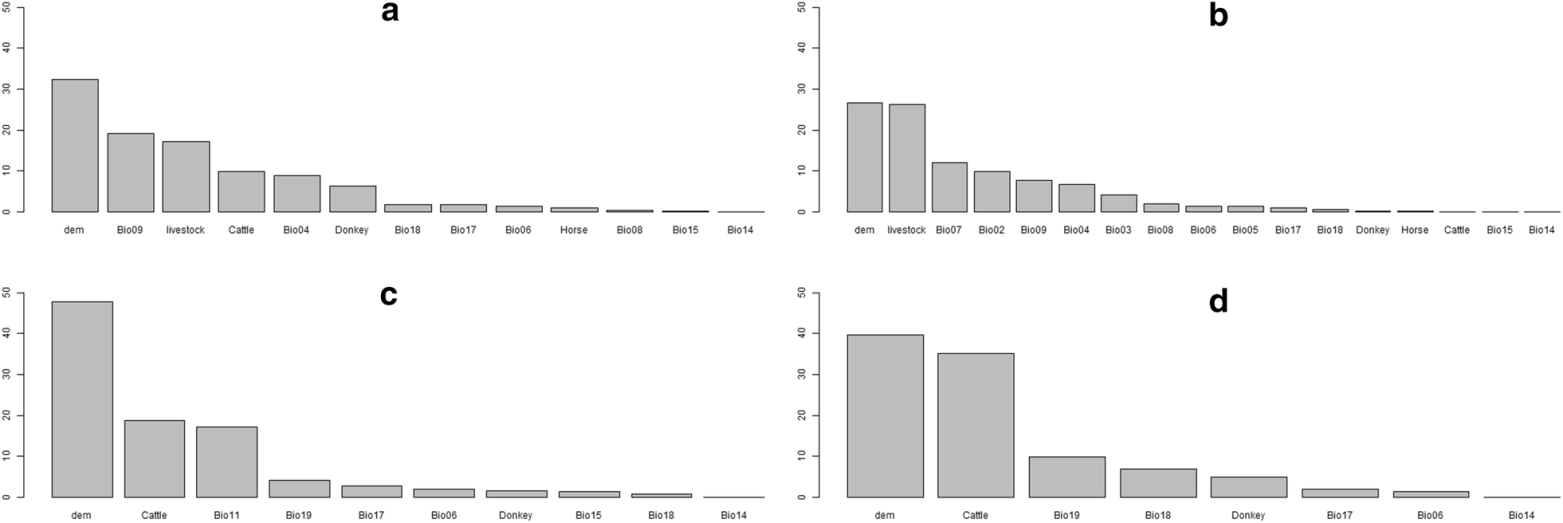
Contribution (%) of each variable to the building of the Maxent models. *C. imicola* (**a**), *C. oxystoma* (**b**), *C. enderleini* (**c**) and *C. miombo* (**d**)

**Fig. 5:**
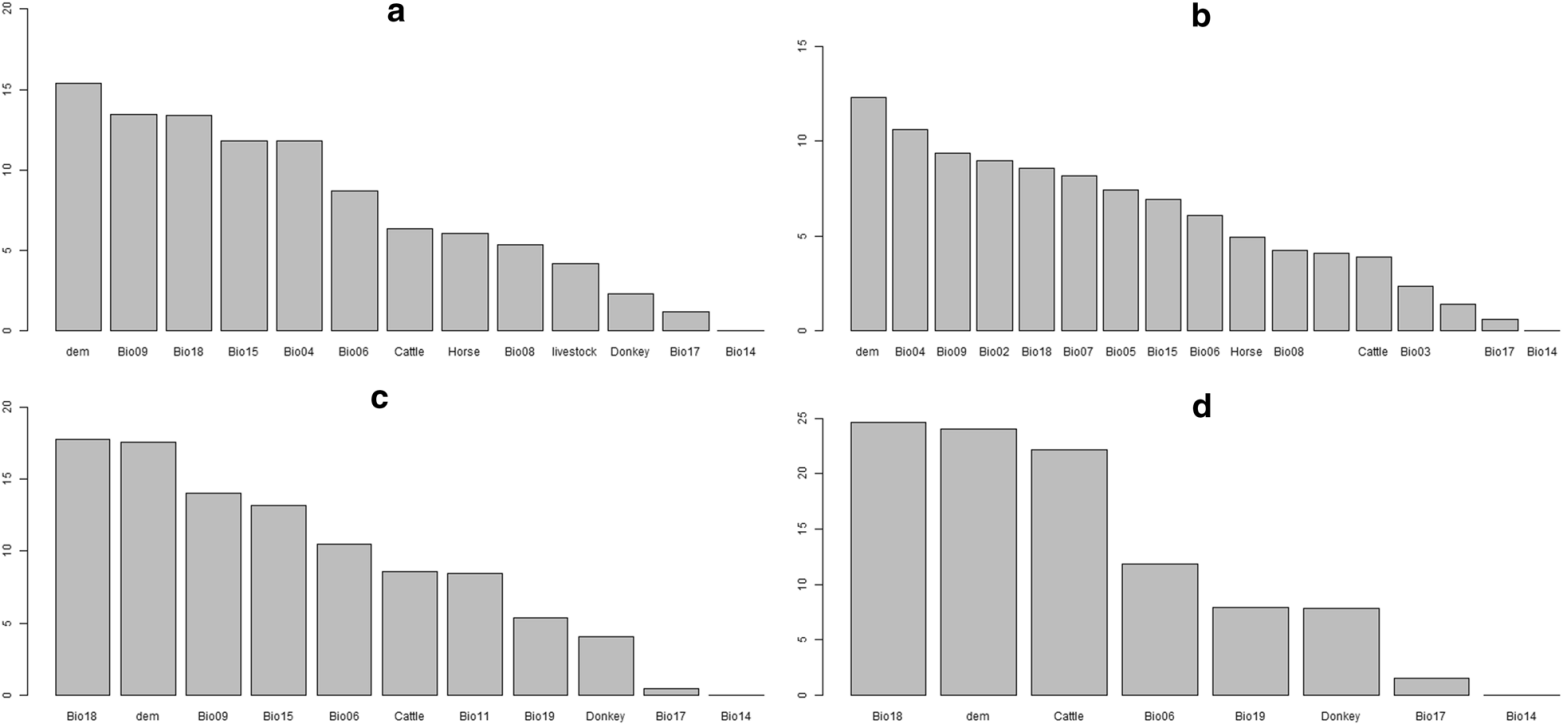
Contribution (%) of each variable to the building of the BRT models. *C. imicola* (**a**), *C. oxystoma* (**b**), *C. enderleini* (**c**) and *C. miombo* (**d**)

Figures 4 and 5 show the contributions of each of the environmental and livestock layers to the habitat suitability of the MaxEnt and BRT models, along with their influence.

For the MaxEnt model, altitude was the most important variable driving *Culicoides* species distribution, all species included (Fig. 4). The other most important variables were the mean temperature of the driest quarter, the cumulative livestock density and temperature seasonality for *C. imicola* (Fig. 4a), the cumulated livestock density, annual temperature range, mean diurnal range and mean temperature of the driest quarter for *C. oxystoma* (Fig. 4b), the cattle density, mean temperature of the coldest quarter, precipitation of the coldest quarter and precipitation of the driest quarter for *C. enderleini* (Fig. 4c) and cattle density, mean temperature of the coldest quarter, precipitation of the warmest quarter, precipitation of the wettest quarter and donkey density for *C. miombo* (Fig. 4d).

Comparatively, for the BRT model, altitude was the most important variable driving *Culicoides* species distribution for two species, *C. imicola* and *C. oxystoma* and the precipitation of the warmest quarter for *C. enderleini* and *C. miombo* (Fig. 5). The other most important variables were the mean temperature of the driest quarter, precipitation of the warmest quarter, precipitation seasonality and temperature seasonality for *C. imicola* (Fig. 5a), temperature seasonality, the mean temperature of the driest quarter, mean diurnal temperature range and precipitation of the warmest quarter for *C. oxystoma* (Fig. 5b), the altitude, mean temperature of the driest quarter, precipitation seasonality and minimum temperature of the coldest month for *C. enderleini* (Fig. 5c), and the altitude, cattle density, minimum temperature of the coldest month and precipitation of the coldest quarter for *C. miombo* (Fig. 5d).

Hence, considering these two models, the most common contributing variables for building them were the altitude derived from digital elevation model (dem), the maximum temperature of the warmest month, the precipitation of the warmest quarter, mean temperature of the wettest quarter, temperature seasonality, the precipitation of the wettest quarter and the livestock density.

## Discussion

Predictive modelling of species geographical distributions based on the environmental conditions of known occurrence sites is a major technique in analytical biology, with applications in conservation and reserve planning, ecology, evolution, epidemiology, invasive-species management and other fields [18–21].

A nationwide entomological sampling campaign enabled the collection of 1,373,929 specimens of the genus *Culicoides* belonging to at least 32 different species, at 96 different sites in 12 out of 14 regions of Senegal. For security reasons in southern Senegal, the Ziguinchor and Sédhiou regions were not visited.

In this study, ecological niche models were developed for four potential BTV vectors (*C. imicola*, *C. oxystoma*, *C. enderleini* and *C. miombo* [23]) using entomological data, climate, altitude variables and livestock density, to assess the effect of bioclimatic, altitude and livestock density variables on habitats suitable for *Culicoides*. The ENFA showed that the presence of BTV vectors was positively correlated to precipitation variables and to most of the livestock densities for all species, whilst the altitude (height) https://en.wikipedia.org/wiki/Digital_elevation_model and most of the temperature indices were negatively correlated to species occurrence. The MaxEnt and BRT models predicted the distribution of *Culicoides* based on the factors selected by ENFA. The two types of models used the same set of variables, but the importance of each of them varied depending on the species. The models predicted the same suitable zones, but with different probabilities of species presence. Each model had an AUC greater than 0.77. Based on the AUC, the MaxEnt was better than the BRT model for *C. imicola*, *C. enderleini* and *C. miombo*.

Temperature and precipitation are well known to be climate parameters that influence density and presence of *Culicoides* [68–72]. In this paper, the maximum temperature of the warmest month, the precipitation of the warmest quarter, the mean temperature of the wettest quarter, temperature seasonality and the precipitation of the wettest quarter were among the most driving factors for *Culicoides* species. In Senegal, the warmest and wettest months are during the rainy season (July–November), which includes the wettest quarter (August–October) and the warmest one (July–September). In addition, previous studies showed that the peak abundance of most *Culicoides* species is observed at the end of the rainy season (September–October) [73].

Furthermore, although each species has its own ecological requirements, any larval habitat could be shared by several ecologically-close species [17, 70]. This might explain the spatial co-occurrence of *Culicoides* species as seen in Fig. 2. Despite the fact that the presence of watercourses was not included as a predictor in our analysis, our model predicted the presence of *Culicoides* around Senegalese watercourses and lakes as expected [70]. Variations in *Culicoides* density are directly related to rainfall, hygrometry and temperatures, which condition the productivity of larval habitats and the spatial dispersion of adults [71, 74–76].

Our observations were consistent with those made by Diarra et al. [23]. In fact, by using two different statistical approaches, Random Forest (RF) and Generalized Linear models (GLM), Diarra et al. [23] showed that rainfall and/or NDVI were the most important variables influencing abundance for the 3 species *C. imicola, C. enderleini* and *C. miombo*. According to Diarra et al. [23], the abundance of *C. oxystoma* was mostly determined by the average rainfall and daily average temperature, that of *C. enderleini* by average precipitation, the Normalized Difference Vegetation Index (NDVI, a proxy for vegetation productivity) and the average daily temperature, that of *C. imicola* was mostly driven by the average precipitation and maximum NDVI, and that of *C. miombo* by NDVI followed by the average precipitation and average night temperature. As vegetation productivity in the Sahel zone is largely determined by climatic conditions, especially rainfall, we can, like Diarra et al. [23], confirm that variations in temperature and precipitation are among the best predictors of *Culicoides* occurrence and abundance. In contrast to Diarra et al. [23], we used new statistical approaches in this study with bioclimatic variables (19) covering a 50-year period, an altitude variable, and six more recent animal density variables. This gave 26 combinations of the four major variables (precipitation, temperature, altitude and livestock).

On the other hand, the ENFA showed that the occurrence *Culicoides* vectors of BTV was negatively correlated with altitude, which was the most important driver according to the MaxEnt and BRT models.

It is known that low-lying areas are often characterized by the presence of watercourses, and dense aquatic vegetation with a particular microclimate, so they are very suitable areas for livestock breeding and the development of arbovirus insect vectors [6, 7, 23, 73, 77]. Studies undertaken in South Korea [78], and in Kagoshima, southern Japan [79] showed the presence and abundance of *Culicoides* spp. in areas mainly characterized by a humid subtropical climate. *Culicoides* presence was also negatively correlated to variables linked to temperature, particularly the maximum temperature of the warmest month and the annual temperature range. Areas with these environmental conditions are predominantly covered by a low vegetation mantle (small bushes and trees). Thus, the Niayes area, where this type of vegetation is predominant, could be a suitable habitat for *Culicoides* species. Moreover, Diarra et al. [73], Fall et al. [80] and Diarra et al. [23] showed that *C. oxystoma* is very frequent and abundant in the Niayes area.

Livestock density was found to be positively associated with the occurrence and abundance of BTV vectors. Other studies [7, 81, 82] pointed in the same direction, showing the very complex relationship between *Culicoides* and their favourite hosts, ruminants and horses. For all species of interest, the Niayes area and the Groundnut basin were found to be the most suitable habitats, predicted with a high relative probability of occurrence (p > 0.7). In fact, both these areas are low-lying altitude and characterized by dense vegetation and high livestock density.

Furthermore, other potential predictor variables could be included in our model: the application of *Culicoides* control strategies, socio-economic status, human population densities, the presence of *Culicoides* biological predators, etc. However, our model effectively described habitat suitability using only altitude, temperature, precipitation and livestock density variables.

## Conclusions

We presented ecological niche models for the BTV vectors, *C. imicola*, *C. oxystoma, C. miombo* and *C. enderleini*, on a nationwide scale in Senegal. This modelling approach allowed us to determine the effect of bioclimatic variables and to generate occurrences of *Culicoides* as risk factors for disease outbreaks. The results from this analysis can be used to (i) improve the quality of BT intervention plans identifying the areas of highest priority for intervention (reducing personnel and equipment costs) and (ii) provide a useful tool for researchers and disease control teams for further studies. Our models represent one of the first essential, albeit laborious, steps towards these future applications.

## Materials and methods

### Study area

**Fig. 6:**
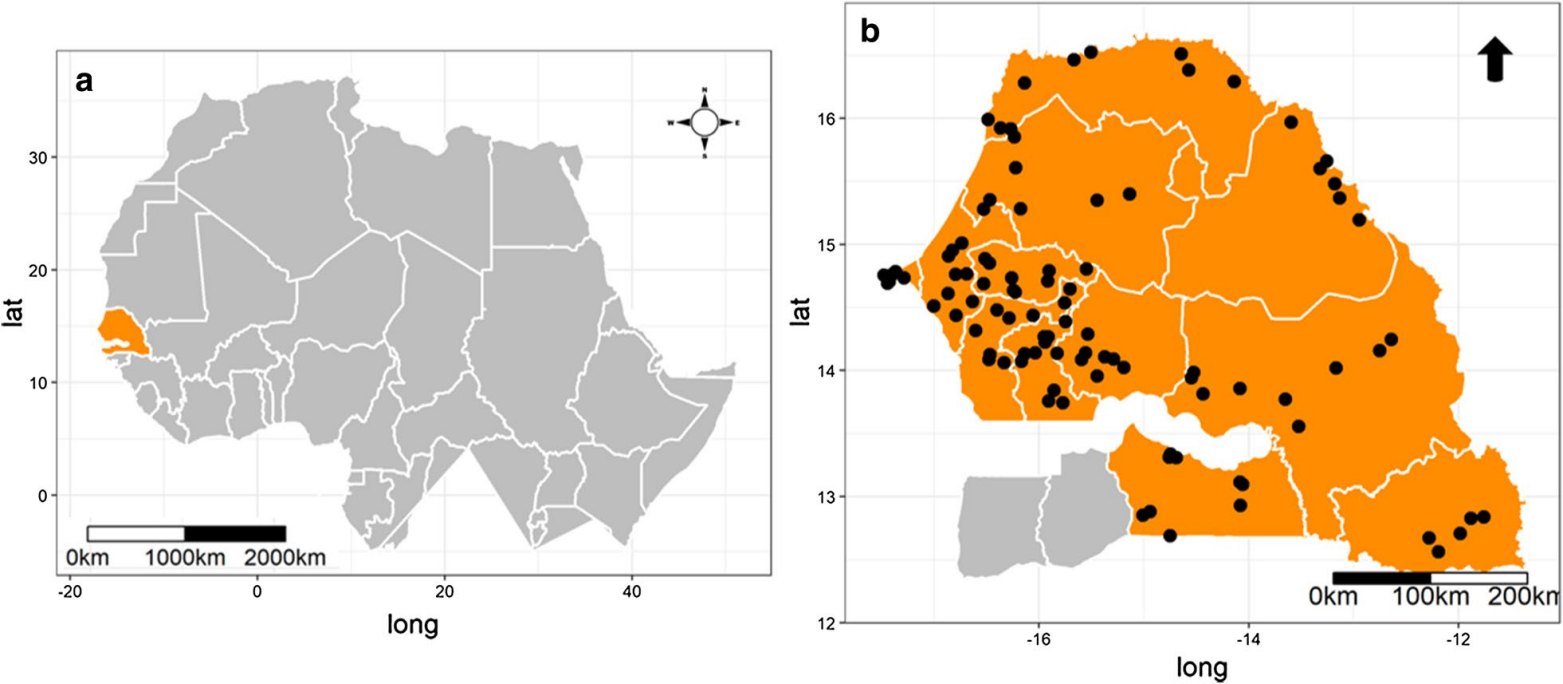
Map of Senegal, a West African country (**a**), with the location of study sites in 12 Senegalese regions (**b**). In yellow, the study area and in grey, the unsampled area

As a part of the nationwide surveillance programme in Senegal in 2012, 108 livestock premises were initially selected (as trapping sites) as follows: 3 departments per region, in 12 of the 14 Senegalese regions and 3 sites per department. The Ziguinchor and Sédhiou regions were excluded for safety reasons. In this study, we only considered data from 96 sites (Fig. 6) which were visited at the end of the 2012 rainy season (in September and October).

### Data collection

#### Entomological data

*Culicoides* specimens were collected on two consecutive nights at each site using Onderstepoort black-light suction traps (Onderstepoort Veterinary Institute South Africa) positioned close to livestock’s enclosures. The geographical coordinates of each site were recorded with a Garmin© hand-held global positioning system receiver (accurate to within 10 m) and projected in UTM Zone 28N. Several identification keys were used depending on the species found and their subgenus or group [83–87]. For species that were difficult to identify, the specimens were dissected and slide-mounted in accordance with the Wirth and Marston technique for observation under a microscope [88, 89].

#### Climatic, environmental and livestock parameters

Several variables (26 in total) were used to implement the model. These were grouped in 4 categories (Table 2): 11 bio-climatic variables related to temperature (Bio01–Bio11); 8 bio-climatic variables related to precipitation (Bio12–Bio19); elevation data (1 variable) and animal density (6 variables).

**Table 2:** Variables, description and code used in the ENFA and MaxEnt niche models

Category of variables	Description	Abbreviation/code
Temperature	Mean annual temperature	Bio01
	Mean Diurnal range [mean of monthly (max temp − min temp)]	Bio02
	Isothermality (Bio02/Bio07)*100	Bio03
	Temperature seasonality (standard deviation * 100)	Bio04
	Maximum temperature of warmest month	Bio05
	Minimum temperature of coldest month	Bio06
	Annual temperature range (Bio05–Bio06)	Bio07
	Mean temperature of wettest quarter	Bio08
	Mean temperature of driest quarter	Bio09
	Mean temperature of warmest quarter	Bio10
	Mean temperature of coldest quarter	Bio11
Precipitations	Annual precipitation	Bio12
	Precipitation of wettest month	Bio13
	Precipitation of driest month	Bio14
	Precipitation seasonality (coefficient of variation)	Bio15
	Precipitation of wettest quarter	Bio16
	Precipitation of driest quarter	Bio17
	Precipitation of warmest quarter	Bio18
	Precipitation of coldest quarter	Bio19
Altitude	Digital elevation model	Dem
Animal density	Cumulative density of horses, cattle, donkeys, goats and sheep	Livestock
	Cattle density	Cattle
	Goat density	Goat
	Sheep density	Sheep
	Horse density	Horse
	Donkey density	Donkey

The bioclimatic data, with a spatial resolution of 30 arc-seconds (~ 1 km), were downloaded from the World Climate [90] website (http://www.worldclim.org/current) and averaged over a 50-year time period between 1950 and 2000 at the same spatial resolution. Elevation data (digital elevation model) were extrapolated from the Moderate Resolution Imaging Spectroradiometer (MODIS) with a spatial resolution of 30 arc-seconds (~ 1 km). Lastly, livestock data (number of head of cattle, small ruminant, horses and donkeys) were extracted from a survey undertaken at department level by the Direction des Services Vétérinaires (DSV), the Senegalese national institute and relevant body for animal health (DSV, 2013, unpublished work). Before being stacked together, the livestock data (6 variables) were rasterized by department with a spatial resolution of 250 m × 250 m. Livestock data were used due their importance for vector life [7, 81]. All layers were resampled at a spatial resolution of 250 m × 250 m using the nearest neighbour method and were stacked in one single spatial layer.

### Modelling habitat suitability

We first carried out an exploratory analysis to identify the variables determining habitat suitability (i.e. the presence of the species). To that end, we used an Ecological Niche Factor Analysis (ENFA) [42, 43], a multivariate approach, similar to PCA, which requires only presence data for the species, in order to explore relationships between occurrence and environmental data. The first axis (marginality axis) of the ENFA is a measurement capturing the dimension in the ecological space conditions where the species is found are far from the global environmental conditions; a large marginality value would imply that the conditions where the species is found are “far” from the overall environmental conditions. In contrast, the second axis (specialization) is a measurement of the narrowness of the niche (ratio of the multidimensional variances of available to occupied spaces). During this procedure, highly correlated variables (with a coefficient of correlation higher than 0.95), and those that did not contribute to the ecological niche, were not used in the statistical analyses.

After selecting the variables, we used the MaxEnt approach [19, 91, 92] to model *Culicoides* presence. MaxEnt is a machine learning technique based, as the term indicates, on the principle of maximum entropy for a probability distribution, given constraints on its momenta:

$$ {\text{Entropy}} = - \mathop \sum \limits_{i} p_{i} \log p_{i} $$

where $p_{i}$ corresponds to species presence probability in the *i*th cell. The method uses presence locations and environmental covariates for all cells in the study area. Data, used to inform the model, define moment constraints on the distribution, while covariates define the mean, variance etc. where species occur. The result is an estimation of the presence probability in each cell.

Like MaxEnt, Boosted Regression Tree Modelling (BRT) [93] is another machine learning technique. The BRT approach developed by Friedman [94] uses two algorithms [30]: regression trees [95] and a boosting technique [96]. Over the past few years, this technique has emerged as one of the most powerful methods for predictive data mining. Some implementations of these powerful algorithms allow them to be used for regression as well as classification problems, with continuous and/or categorical predictors. Basically, the algorithm computes a sequence of simple trees, where each successive tree is built from the prediction residuals of the preceding tree.

Entomological data (for each species) were randomly divided into two samples, training and testing, using the K-fold cross-validation method. Accordingly, the original data were divided into k samples, then one of the k samples was selected as the validation set and the other k − 1 samples were the learning set. The performance score was calculated, and the operation was repeated by selecting another validation sample from the k − 1 samples that had yet used for model validation. The operation was repeated k times so that ultimately each sub-sample had been used exactly once as a validation set. The mean of the k mean squared errors was finally calculated to estimate prediction error. In this work we used k = 5.

Model performance was tested using the Area under the ROC curve (AUC), a plot of sensitivity against specificity measuring the ability of the model to discriminate between sites where a species was present (y = 1), as opposed to where it is absent (y = 0) [97–99]. AUC values range from 0 to 1; an AUC value higher than 0.8 indicates robust performance of the model. Statistical analysis and modelling were performed with R [100] using the following R-packages: adehabitatHS [101] for ENFA computation, dismo [102, 103] for MaxEnt and GBM [93] for BRT modelling.

## Data Availability

All relevant data are presented or refer to their publicly available sources in the main text of the paper. Entomological data supporting the conclusions of this article are already published in Diarra et al. [23]. The bioclimatic data (Bio01**–**Bio19) used are available at the World Climate website: http://www.worldclim.org/current and the digital elevation model layer via the PALE-Blu data archive website in https://www.edenextdata.com/?q=content/modis-1km-digital-elevation-model-and-landwater-mask-v5

## Abbreviations

ENFA: ecological niche factor analysis
AHS: African horse sickness
AHSV: African horse sickness virus
BT: bluetongue
BTV: bluetongue virus
AUC: area under the ROC curve
